# Impact of a Generated Magnetic Field at the Schumann Resonance Frequency on Skin Blood Perfusion and Peripheral Pulse Amplitudes

**DOI:** 10.7759/cureus.83182

**Published:** 2025-04-29

**Authors:** Harvey N Mayrovitz

**Affiliations:** 1 Medical Education, Nova Southeastern University Dr. Kiran C. Patel College of Allopathic Medicine, Davie, USA

**Keywords:** arterial pulses, finger pulses, heart rate variability, laser doppler, magnetic effects, skin blood flow, time-varying magnetic fields

## Abstract

A pilot study was conducted in six young adult male volunteers (25.8±1.3 years) to determine if there was evidence of an effect of a locally applied magnetic field at the Schumann Resonance fundamental frequency of 7.8 Hz. The field was generated by magnets attached to the shaft of an electronically controlled motor and applied for 20 minutes to the wrist region near the radial and ulnar arteries. The alternating magnetic field was activated after the subject had been supine and resting for 20 minutes. Skin blood perfusion (SBF) was measured on the thenar eminence via laser Doppler flux (LDF), and finger pulses were measured by photoplethysmography (PPG) on the index finger as indicators of localized hemodynamics and heart rate variability (HRV) determined as a measure of possible central effects. These measurements were done before magnet activation and after its deactivation, so each subject served as their own control. No separate sham procedure was used. Results showed that LDF decreased by 23.6±22.5% (p<0.05) and PPG pulse amplitude decreased by 35.5±18.7% (p<0.05) from pre- to post-magnetic field exposure based on a non-parametric Wilcoxon signed-rank test. There was no statistically significant change in HRV, although this study did not evaluate the possibility of a different systemic effect. These pilot findings are based on a minimal number of subjects but provide new data and lay the groundwork for a more extensive investigation of this process, which seems warranted based on the present findings.

## Introduction

Schumann Resonances (SR) [1] are associated with global lightning discharges that result in naturally occurring extremely low-frequency electromagnetic fields with a fundamental frequency spectral peak of about 7.8 Hz and other less intense peaks at about 14, 20, and 26 Hz and beyond. The 7.8 Hz fundamental frequency corresponds to a wavelength of about 38,460 km. Lightning discharges act as vertical radiators, causing traveling electromagnetic waves that circle the globe within the Earth-ionosphere waveguide [2]. A resonant fundamental standing-wave peak occurs via constructive interference of these waves when the wavelength of the electromagnetic waves generated by the lightning is close to the Earth’s circumference, at about 40,000 km. There are reports of correlations between SR frequencies and cardiovascular parameters, including an association between hospital admissions for cardiovascular events [3]. Further, ambulatory systolic, diastolic, and mean blood pressure measured over seven days were reported to be lower during enhanced SR activity when using a threshold above two picoTesla (pT) [4]. Other examples of the effects of SR activity on cardiovascular parameters include the increased spectral power of heart rate variability [5] and generalized parasympathetic synchronization [6]. An extensive review of the reported SR effects suggests that the brain receives the very low frequencies associated with SR and responds to them, triggering various biological effects [7]. Recent in vitro studies of rat myocytes indicated that artificial excitation at an SR frequency of about 7.8 Hz altered calcium dynamics and caused spontaneous contractions to diminish and then cease after 25 minutes of application [8]. Although it is unknown if a similar effect will occur in vascular smooth muscle, the findings above prompted us to hypothesize that local excitation at such a frequency might impact blood flow via the withdrawal of some the sympathetic tone of blood vessels or via direct effects within or near the magnetically exposed tissue. The primary goal of this pilot study was to explore this concept and test this hypothesis via measurements of skin blood perfusion and peripheral pulses before and immediately following localized exposure to generated magnetic fields at the fundamental SR frequency. Its importance lies in the basic information that might be forthcoming regarding a long-debated biological and physiological issue. The foundation for future research possibilities may evolve if there are indications of such interactions.

## Materials and methods

Subjects

A convenience sample of six male volunteers aged 25.8±1.3 years (mean±SD), recruited from the first- and second-year medical students, participated in this research after signing a Nova Southeastern University Institutional Review Board (IRB) approved consent form (approval no. NSU #2021-2). Entry requirements were (1) the ability and agreement to lie supine without significant movements for up to 60 minutes, (2) no history of cardiovascular or neurological conditions, (3) nonsmoker and (4) willingness to forgo caffeinated beverages on the day of the experiment. People with diabetes mellitus were excluded from participation. The absence of cardiovascular or neurological conditions was based on the participants’ self-report.

Procedures

**Figure 1:**
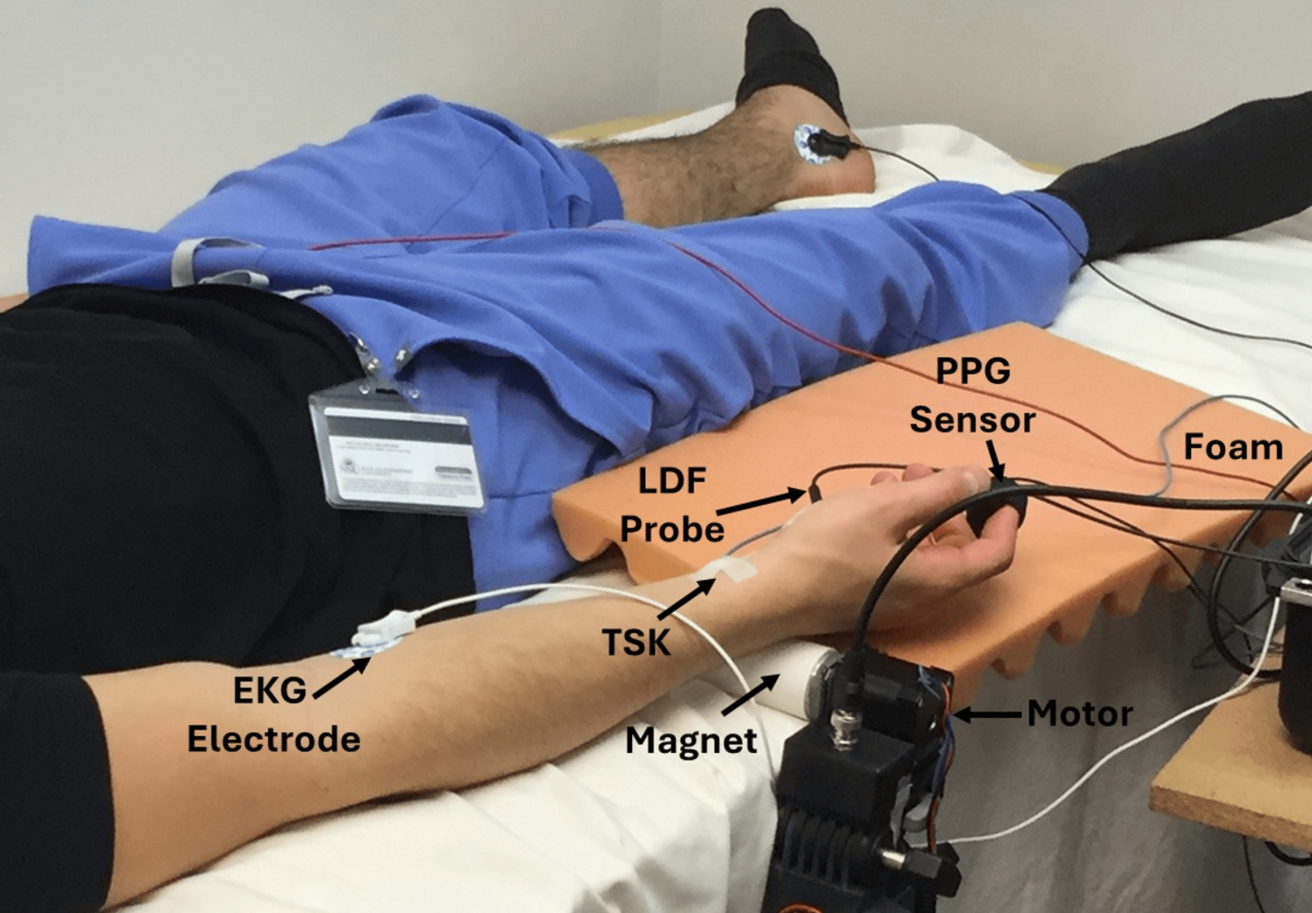
Experimental setup The arm and hand rest on a padded exam table and supportive foam material, with the body part just in contact with the protective shell encasing the rotating magnet. Skin blood perfusion is measured with a laser Doppler probe (LDF). Finger pulses are measured with a photoplethysmographic (PPG) sensor, and skin temperature is measured at the wrist.

Subjects arrived at an experimental room at a time and day previously scheduled and remained in a supine position on a padded examination table. The subject’s right hand was supported and positioned over the outer shell of a self-designed and built magnetic pulse excitation device, as shown in Figure 1.

The hand was supported by soft foam and not in direct contact with the outer shell of the magnet. The subject was told that the magnet device might or might not be activated sometime during their participation. 

The body part was exposed to a magnetic field generated by magnets attached to the motor shaft that was electronically rotated at a frequency of 7.8 Hz. The features and properties of the magnetic device are described subsequently. Measurements that included skin blood perfusion via laser Doppler flux (LDF), finger pulses via photoplethysmography (PPG), skin temperature (TSK), and a three-lead electrocardiogram (EKG) were made with the hand resting on and supported by soft foam with the skin just touching the outer surface of the shell encasing the rotating magnets. Five minutes after positioning the hand, data recording started for 45 minutes. For the first 20 minutes, the magnetic field-generating device was not activated. At the 20-minute mark, it was turned on to generate magnetic pulses at a frequency of 7.8 Hz for 20 minutes. It was turned off at the end of the 20-minute activation time, and data was recorded for an additional five minutes. The subject was not aware of when the device was activated or deactivated. During this time, the room lights were dimmed, talking was prohibited, and other disturbances were minimized. At the end of the recording interval, the subjects were asked if they knew if and when the magnet had been activated. None of the subjects reported knowledge of this. Thereafter, the subject's blood pressure was measured in their left arm with an automatic blood pressure system (HEM-711, Omron Healthcare, Sunrise FL, USA).

Measurements

**Figure 2:**
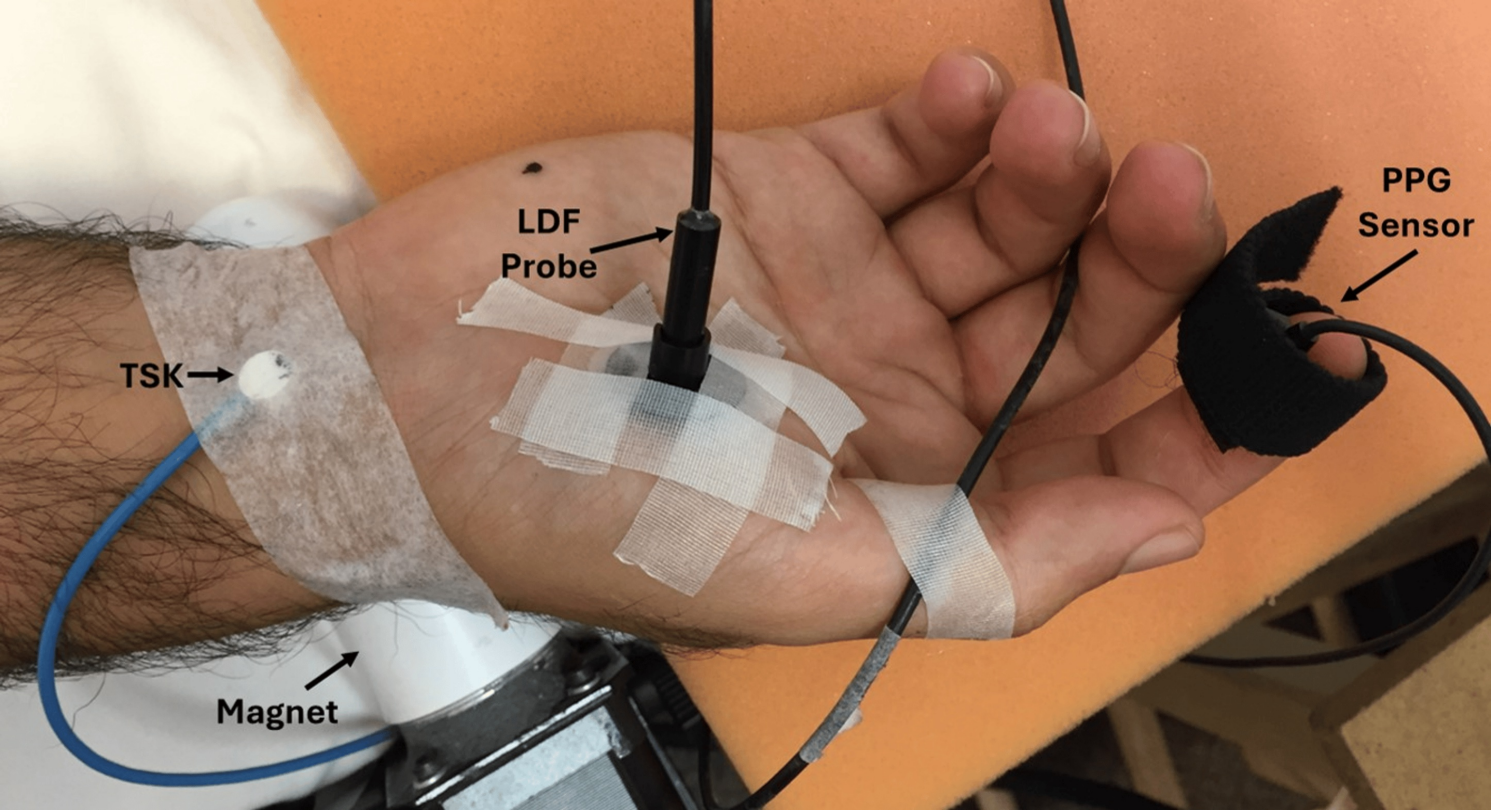
Placement of measurement sensors LDF is measured in the center of the right hand's thenar eminence, and PPG is measured on the index finger of the same hand. The hand is shown resting above the surface of the rotating magnet's protective shell. Skin temperature (TSK) is measured at the wrist. LDF, laser Doppler flowmetry; PPG, photoplethysmography

LDF was measured with a multifiber laser integrating probe (Moor DP7A, Moor Instruments, Wilmington DE, USA) attached to the middle of the thenar eminence of the right hand, as shown in Figure 2.

The probe was connected to a laser Doppler measuring system (Laserflo Blood Perfusion Monitor Model BPM2, Moor Instruments, Devon, UK). The recorded blood perfusion or flux is the product of the red blood cell (RBC) concentration and RBC velocity. The measurement uses a low-intensity laser light signal transmitted into the skin to a depth of about 1-2 mm [9]. The Doppler-shifted return signal contains information about the speed and number density of moving RBCs, which is processed to yield a parameter, RBC perfusion or RBC flux, proportional to the blood flow. The system is calibrated using a motility standard provided by the company. LDF is expressed as relative arbitrary units (au) since it cannot be directly expressed in blood flow units but is widely used to measure skin blood perfusion [10-13]. The PPG pulse was recorded from the index finger of the same hand using a matched infrared emitter and photodiode sensor (TSD200, Biopac Systems, California, USA) that was gently but securely secured to the finger with Velcro. The system works with an infrared excitation of 860 ± 60 nm with a detected wavelength of 800 nm. Blood pulses in the finger tissue modulate the infrared, causing changes in the sensor's resistance, producing a time-varying output voltage reflecting these pulsations.

The PPG sensor output was coupled to a PPG amplifier (PPG100C, Biopac Systems, California, USA) set to a gain of 50, a low-pass filter of 10 Hz, and a high-pass filter of 0.05 Hz. EKG electrodes were connected to an EKG amplifier (EGC100C, Biopac Systems Inc., CA, USA) to record EKG lead I. Skin temperatures at the anterior wrist were measured by a surface sensor (SST-1, Physitemp, New Jersey, USA) secured to the skin using paper tape (3M Micropore, 3M Company, Minnesota, USA) and connected to a control box (Thermalert, Model TH-8, Physitemp, New Jersey, USA). The outputs of the PPG amplifier, laser Doppler system, skin temperature system and EKG amplifiers were coupled to an analog/digital conversion device (DataQ Instruments model DI-720, Ohio, USA) and each channel sampled at 1000 samples per second using Windaq recording and playback software (DataQ Instruments, Ohio, USA) and the output channels displayed and recorded on a laptop computer. 

Magnetic pulse generation

**Figure 3:**
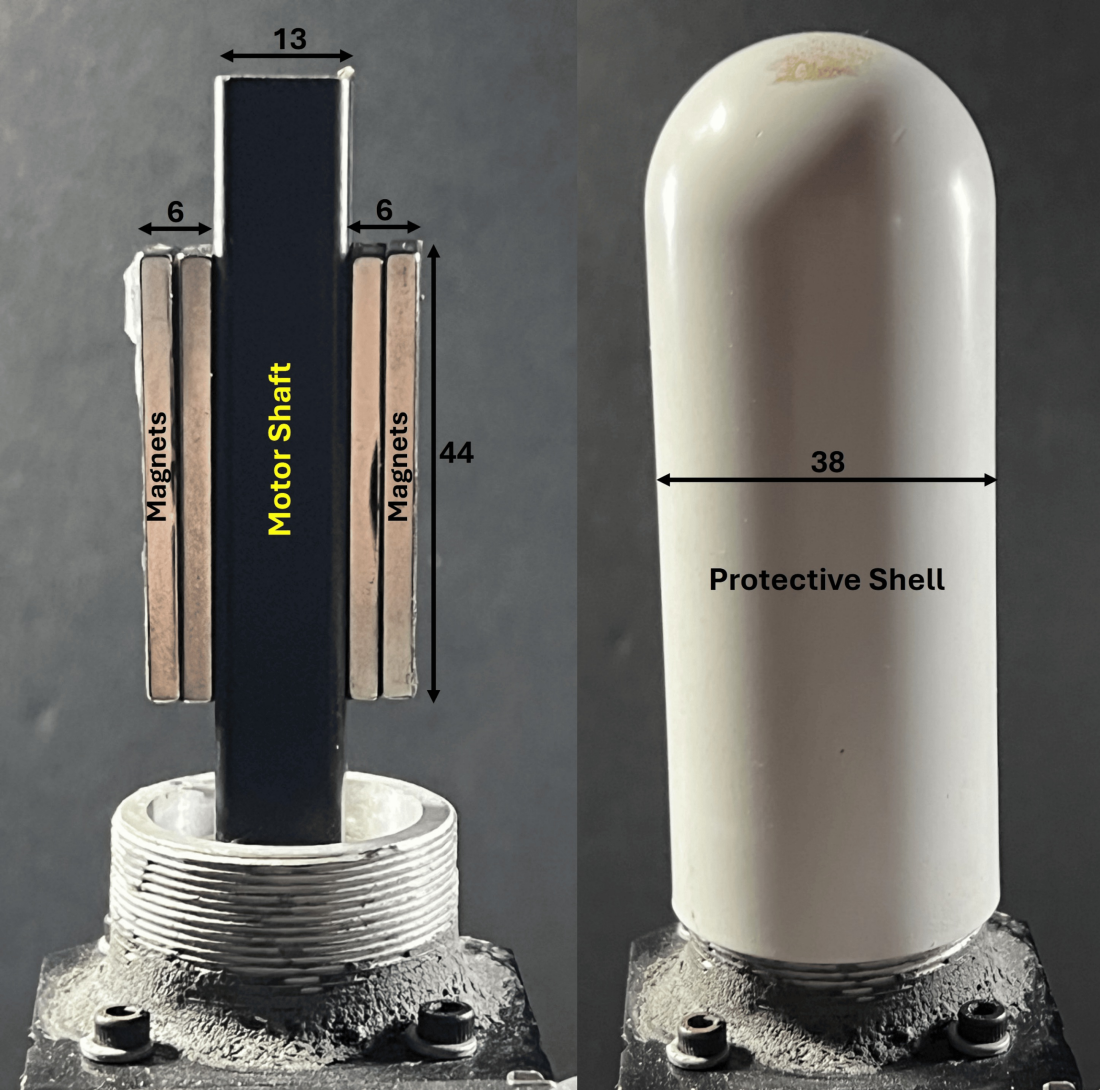
Magnetic pulse generation Two Neodymium N50 magnets, the rotation of which is electronically controlled, are affixed to each side of the motor shaft. The interior is covered with a protective shell for use with a subject. All dimensions are in mm.

The system consists of an electronically controlled motor with neodymium block magnets (K & J Magnetics, N52, Pennsylvania, USA) attached to both sides of its shaft, as illustrated in Figure 3.

**Figure 4:**
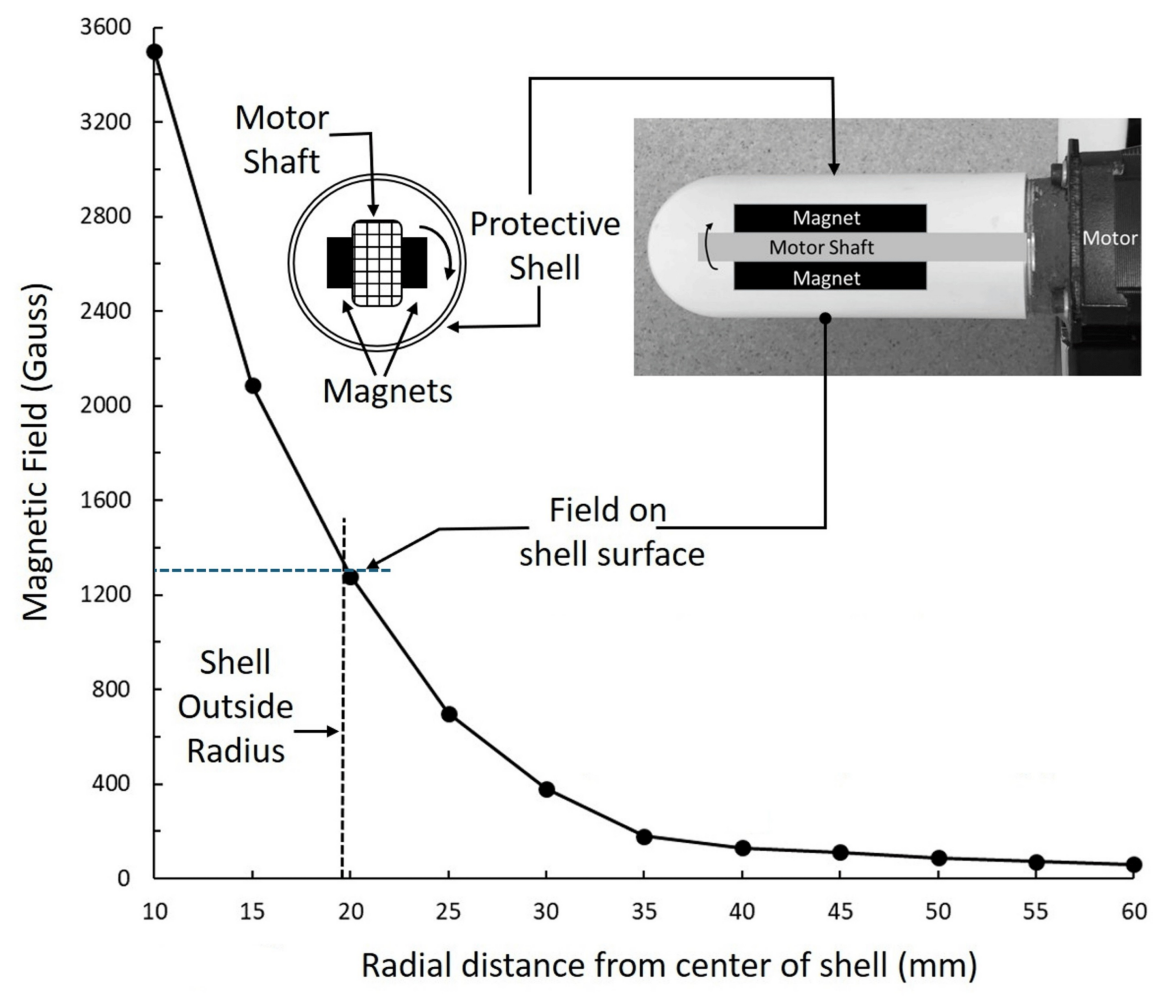
Magnetic field of the rotating magnets The magnetic field on the outer magnet's surface is 3500 Gauss, and on the protective shell's outer surface, it is 1300 Gauss.

When used by the subjects, the magnets and shaft are covered by a protective shell with an outer diameter of 38 mm. The perpendicular magnetic field at the surface of the outer magnet at its center is 3500 Gauss (0.35 T), which reduces to 1300 Gauss (0.13 T) at the surface of the protective shell, as shown in Figure 4.

**Figure 5:**
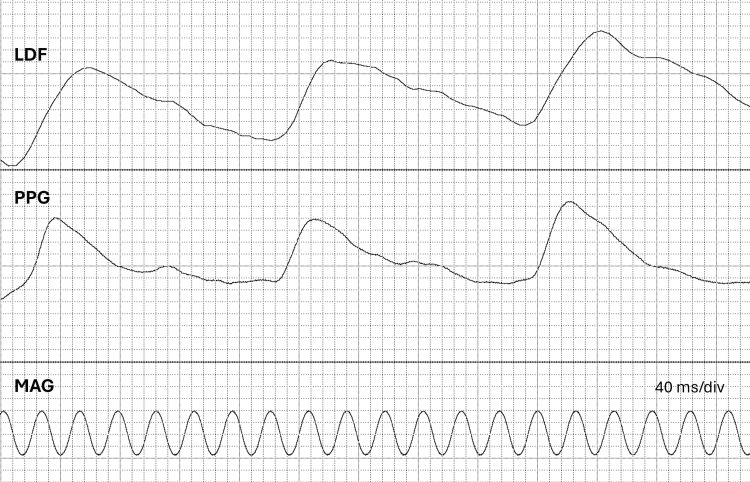
Magnetic field and measured parameters The figure illustrates the magnetic field pattern (MAG), simultaneously measured hand skin laser Doppler flux (LDF), and finger pulse photoplethysmography (PPG) signals. The amplitudes are uncalibrated in this visual.

The measurements were made with a Gaussmeter (Walker Scientific, MG-3A, Massachusetts, USA). The motor speed was controlled electronically to cause the shaft to rotate at a speed that produces a time-varying magnetic field at 7.8 Hz. Figure 5 shows an example of the magnetic field pattern and three cycles of simultaneously measured LDF and PPG signals.

**Figure 6:**
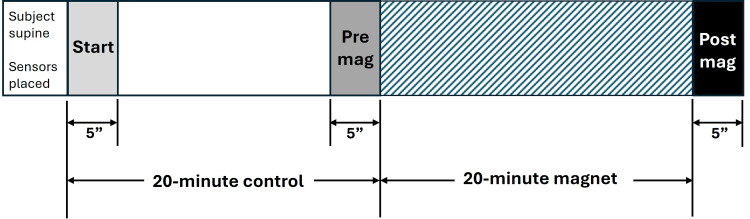
Temporal experimental sequence Average values of LDF and PPG over the indicated five-minute intervals were determined. Two intervals were during the control interval (start and pre-magnet), and one was immediately after the rotating magnetic field was deactivated (post-magnet). LDF, laser Doppler flux; PPG, photoplethysmography

The temporal course of the experimental procedure is summarized in Figure 6.

Analysis

The average skin blood perfusion in the hand was determined as the average LDF during three five-minute intervals: at the start of recording, five minutes before activating the rotating magnetic field, and five minutes immediately following its deactivation. The PPG pulse amplitude was determined during the same five-minute intervals. It was determined by measuring the PPG pulse amplitude for all pulses within these five-minute intervals and calculating their average value. On average, for the group, this included 318±8 pulses (mean±SD). Heart rate variability (HRV) parameters were determined during the five minutes at the end of the control period and during the five minutes immediately after deactivating the magnetic field. This was done by detecting the recorded EKG's R-waves and then determining the peak-to-peak duration using software (Advanced CODAS Analysis Software, DataQ Instruments, Ohio, USA). The adequacy of the automated peak detection process was inspected visually, and any missing or added detections were manually corrected. The time series of consecutive RR intervals was then processed for each of the two five-minute segments using dedicated software for HRV analysis (Kubios HRV standard v3.1.0, Kubios Oy, Finland). Spectral power in the three standardized frequency bands was determined for each interval [14]. These were the very low-frequency band (VLF, 0.003-0.04 Hz), the low-frequency band (LF, 0.04-0.15 Hz), and the high-frequency band (HF, 0.15-0.4 Hz). Power was expressed in units of ms^2^ and, when used, the power spectral density (PSD) in units of ms^2^/Hz. Skin temperatures and heart rates were determined for 30 seconds before activating the magnetic field and 30 seconds immediately after deactivation. All pre- and post-magnet activation comparisons were assessed using the nonparametric Wilcoxon signed-rank test, with a p-value<0.05 assumed to represent a statistically significant difference.

## Results

Participant data

Table 1 summarizes the demographic and other data for each individual in the group.

**Table 1:** Participant data (n=6) Blood pressures were measured at the end of the procedure with each subject supine. Heart rate and wrist skin temperatures were measured and averaged during the 30 seconds before magnetic field activation (pre-magnet) and the 30 seconds immediately after its deactivation (post-magnet). No statistically significant differences existed between pre- and post-heart rate or skin temperature. BMI, Body mass index

					Blood pressure		Heart rate		Skin temperature	
Subj	Age (years)	Height (m)	Weight (kg)	BMI (kg/m^2^)	Systolic (mmHg)	Diastolic (mmHg)	Pre-magnet (bpm)	Post-magnet (bpm)	Pre-magnet (^o^C)	Post-magnet (^o^C)
1	28	1.73	69.2	23.6	117	67	52.3	64.5	32.3	31.8
2	25	1.72	76.5	25.8	126	74	61.6	59.4	35.4	35.4
3	24	1.63	64.7	24.6	121	78	72.6	58.8	35.1	35.5
4	26	1.70	71.4	25.1	120	70	72.1	79.8	32.4	32.0
5	26	1.80	82.1	25.7	134	82	64.8	67.5	35.0	34.8
6	26	1.73	75.9	25.8	120	70	51.3	57.1	36.2	36.4

Group averages (mean±SD) of the participants for age, body mass index (BMI), systolic and diastolic blood pressure were 25.8±1.3 years, 25.1 kg/m^2^, 123±6.1 mmHg, and 73.5±5.6 mmHg, respectively. Blood pressures were measured at the end of the procedure while the subject was supine. Heart rate and skin temperatures were measured and averaged 30 seconds before activating the magnet and 30 seconds after deactivating the magnet. Pre- and post-magnet activation heart rates were 62.5±9.3 bpm and 64.5±8.4 bpm, with the pre-post difference being insignificant (p=0.753) and Cohens-d of 0.225. Similarly, pre- and post-skin temperatures were 34.4±1.6 and 34.3±1.9, respectively, with pre-post differences being insignificant (p=0.496) and Cohens-d of 0.057.

Blood perfusion in the skin

**Figure 7:**
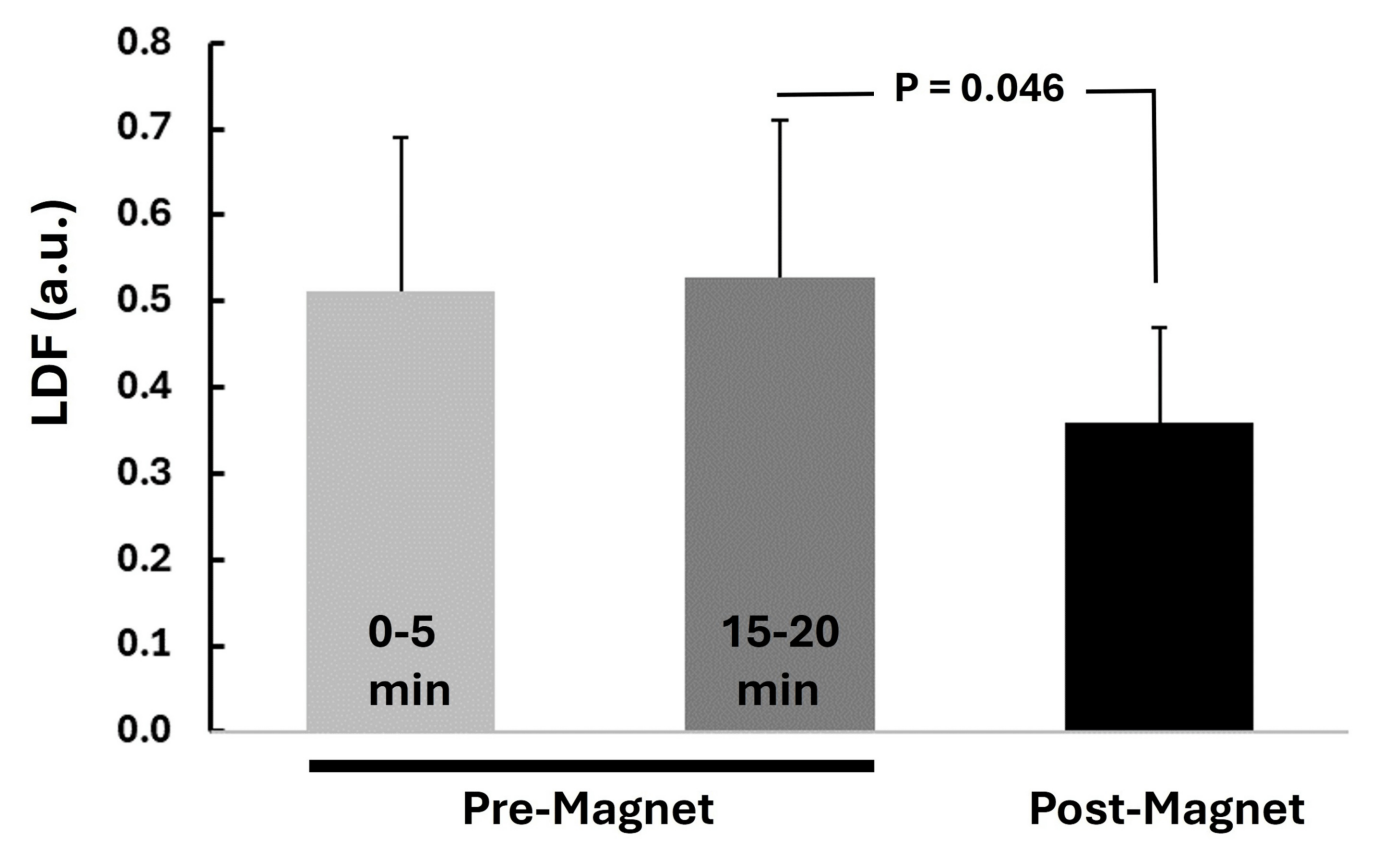
LDF response to 20 minutes of magnetic field exposure The graph indicates the five-minute average values during the pre-magnet control interval at the start (0-5 mins), at the five-minute interval just before activating the rotating magnet (15-20 mins), and at the five-minute interval immediately after deactivating the magnet field (post-magnet). Error bars are standard errors of the mean (SEM). There was no statistically significant difference between the start and pre-magnet (p=0.753). LDF, laser Doppler flux

Figure 7 summarizes the main results for the impact of the 20-minute application of the 7.8 Hz magnetic field on the skin blood perfusion as measured by LDF.

The pre-magnet activation LDF, measured as the average±SD five minutes before magnet activation, was 0.526±0.499 au. During the five minutes after the magnetic rotating field was deactivated, the value significantly decreased to 0.399±0.298 au, p=0.046. Physiologically, this reduction represents a 24.1% decrease in the average blood perfusion that may have been caused by a localized vasodilation attributable to the magnetic intervention. The pre-mag value did not differ from the five-minute average measured at the start of the control interval, which was 0.512±0.435 au, p=0.753. The average percentage reduction in the LDF from pre- to post-magnetic field activation was 23.6±22.5%.

PPG pulse amplitude

**Figure 8:**
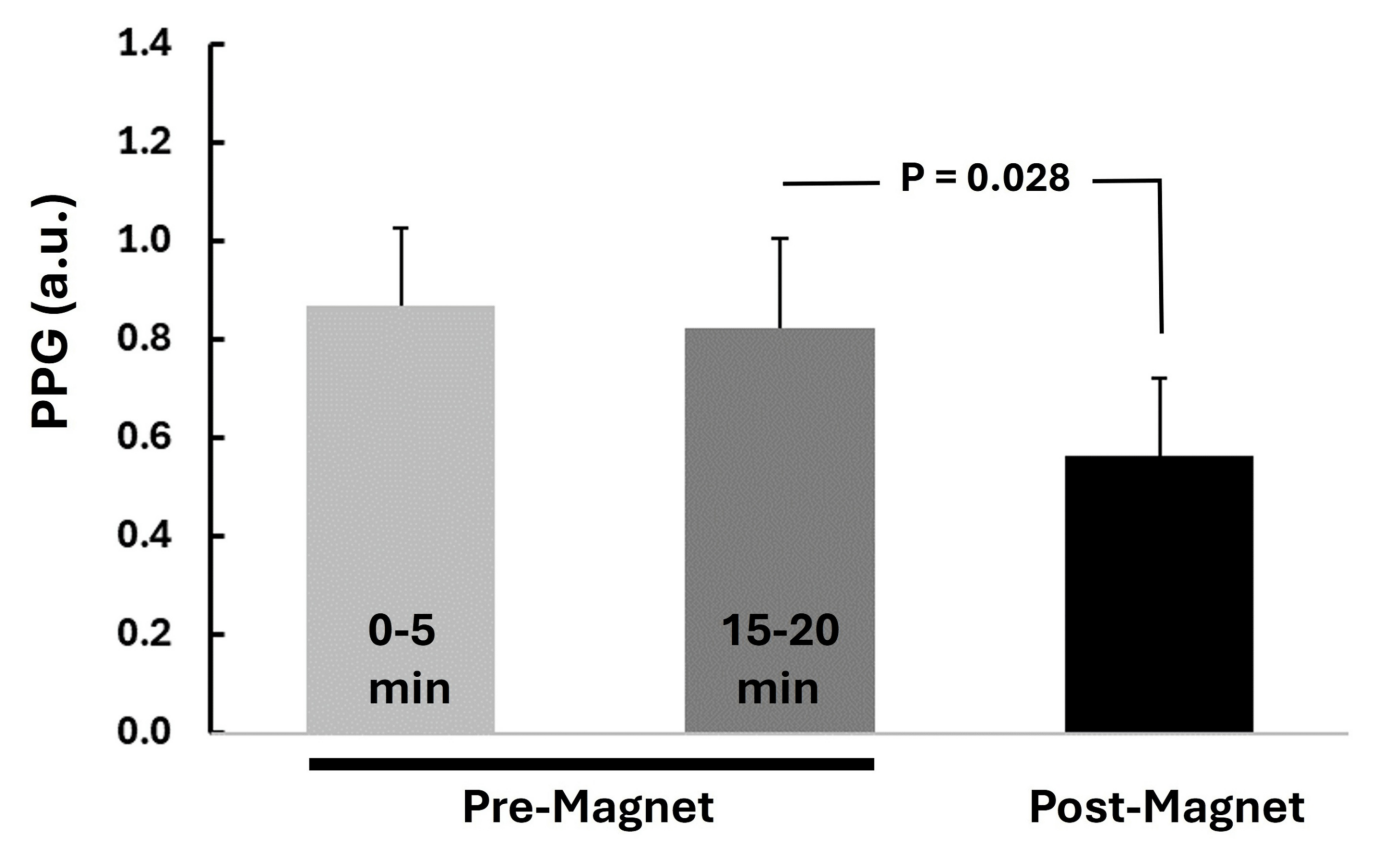
PPG response to 20 minutes of magnetic field exposure The graph indicates the five-minute average values during the pre-magnet control interval at the start (0-5 mins), at the five-minute interval just before activating the rotating magnet (15-20 mins), and at the five-minute interval immediately after deactivating the magnet field (post-magnet). Error bars are standard errors of the mean (SEM). There was no statistically significant difference between the start and pre-magnet (p=0.600). PPG, photoplethysmography

Figure 8 summarizes the results of the impacts of the 20-minute application of the 7.8 Hz magnetic field on the PPG pulse amplitude as measured on the index finger.

The pre-magnet activation PPG pulse amplitude, measured as the average±SD during the five minutes before magnet activation, was 0.822±0.454 au. Five minutes after the magnetic rotating field was deactivated, the value significantly decreased to 0.565±0.387 au, p=0.028. The pre-mag value did not significantly differ from the five-minute average measured at the start of control, which was 0.868±0.392 au, p=0.600. The average percentage reduction in the PPG pulse amplitude from pre- to post-magnetic field activation was 35.5±18.7%. This PPG amplitude reduction would be consistent with the arteriolar vasoconstriction proximal to the site of the PPG measurement.

Heart rate variability (HRV) parameters

**Figure 9:**
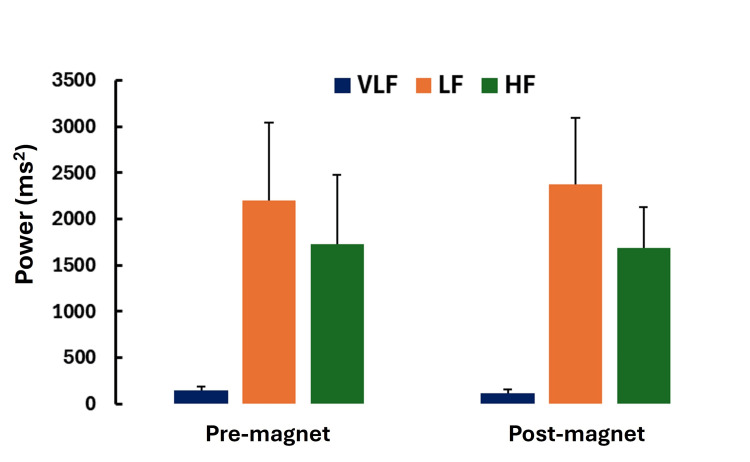
HRV spectral power distribution Absolute power within each of the spectral bands (VLF, LF, and HF) was determined during the five minutes before magnet activation (pre-magnet) and during the five minutes immediately after magnet deactivation (post-magnet); there was no significant difference in pre-post power in any of the frequency bands. Error bars are standard errors of the mean (SEM). HRV, heart rate variability; VLF, very low frequency; LF, low frequency; HF, high frequency

Figure 9 summarizes the absolute power within each spectral band determined during the five minutes before magnet activation and the five minutes immediately after magnet deactivation.

There was no significant difference in power between pre- and post-magnetic field activation in any frequency band. The least total power was within the VLF band, averaging 131 ms^2^, and was significantly less than the power in the LF band (p=0.028) and the HF band (p=0.28). Because the VLF band is associated with slow-changing physiological processes, the total power within this band, for short sampling intervals, is expected to be low compared to the other bands that represent more rapidly changing physiological processes. The most power was within the LF band, averaging 2290 ms^2^. However, there was no significant difference between the average HF power (1709 ms^2^) and LF power (p=0.917).

**Figure 10:**
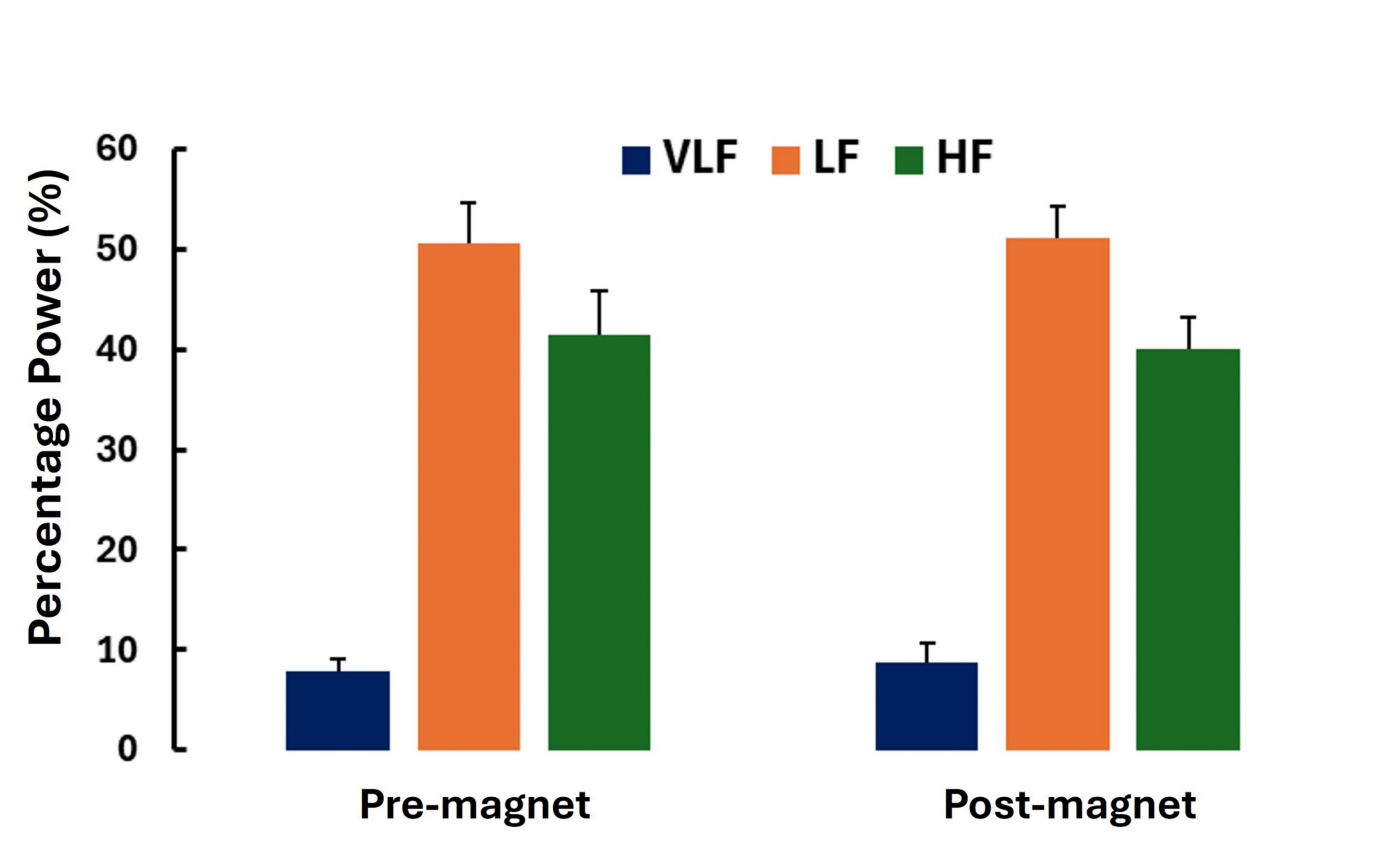
Percentage power by frequency band Percentage power within each of the spectral bands (VLF, LF, and HF) was determined during the five minutes prior to magnet activation (pre-magnet) and during the five minutes immediately after magnet deactivation (post-magnet). There was no significant difference in pre-post power percentages in any frequency bands. Error bars are standard errors of the mean (SEM). VLF, very low frequency; LF, low frequency; HF, high frequency

Figure 10 summarizes the power distribution among the frequency bands during the five-minute interval before magnet activation and during the five minutes immediately post-magnet deactivation.

Considering the power distribution, which is the percentage of total power within each frequency band, potential confounding effects of variability in total power among subjects can be partially minimized. There was no significant difference in this power distribution between pre- and post-magnetic field activation. The percentage of power (expressed in ms^2^ with mean±SD) in the VLF, LF, and HF bands before magnetic field activation was 7.8±2.9, 50.7±10.2, and 41.5±10.6, respectively. The corresponding values post-deactivation were 8.7±4.7, 51.3±7.7, and 40.0±7.9.

## Discussion

The present work builds on a variety of prior investigations that have addressed broad questions of the impact of electromagnetic energy on physiological processes, in which the energy form could be naturally occurring, and might affect blood pressure [15], heart rate [16], or HRV [17,18]. The specific focus in the present pilot study was on the possible impact of a pulsating magnetic field at the fundamental frequency of the SR. More specifically, the primary area of interest was whether an exposure to the artificially generated 7.8 Hz magnetic field would impact cardiovascular processes exemplified by peripheral blood perfusion and pulses, which until now had not been studied. Another study component was to assess the impact on centrally related processes characterized by changes in HRV, since these have been reported to occur in conjunction with SR [5]. 

The main findings showed a reduction in skin blood perfusion in the hand and peripheral finger pulse PPG amplitude when both parameters were assessed for five minutes immediately after 20 minutes of sustained localized magnetic pulse exposure at 7.8 Hz. The magnetic pulses had no apparent effect on the spectral measures of HRV.

The finding of reduced perfusion was consistent with reports that had indicated a reduction in perfusion when tissues were exposed to static magnetic fields in animals [19], and an increase in the vascular tone of some blood vessels [20,21] that would be associated with reduced blood perfusion. In some studies, applying static magnetic fields has also decreased blood perfusion in the human skin [19,22,23]. In contrast, other animal experiments have indicated increased blood perfusion when exposed to static magnetic fields [24]. On the other hand, other human studies have reported varied effects of static magnets, ranging from no effect on blood perfusion [25,26] to an increase [27] or alterations in flow-related spectral dynamics [28]. 

Data on the effects of pulsating magnetic fields at the frequency herein, used on skin blood perfusion or pressure pulses, is scarce. A small study used pulse-modulated 27.1 MHz excitation and assessed forearm skin blood perfusion during a single treatment session in nine volunteers, and a significant perfusion increase was reported [29]. Using the same 27.1 MHz treatment modality in 15 diabetic patients with lower extremity ulcers increased peri-ulcer skin blood perfusion [30]. Contrastingly, treatment of people with diabetes using a 12 Hz pulsed electromagnetic field (PEMF) failed to impact foot skin blood perfusion significantly [31]. Foot skin blood perfusion was also not affected by 30-minute exposures to 10 Hz PEMF in healthy persons [32]. Whole body exposure of 10 volunteers to a 60 Hz magnetic field for 45 minutes also failed to impact skin blood perfusion [33]. 

In considering possible explanations for the observed decrease in skin blood perfusion, the impact of the time-varying field on local blood vessels and nerves should be considered. With the hand positioned as in Figure 2, the radial and ulnar arteries and the median ulnar nerves are exposed to the magnetic field. Since the skin blood perfusion measured with the LDF probe on the thenar eminence is supplied by a superficial branch of the radial artery, field-induced vasoconstriction of either artery or its branches could account for the decrease in perfusion. Similarly, such field-induced vasoconstriction could account for decreased finger pulse amplitude. Vasoconstriction of these vessels might be explained by some reported impacts of pulsed electromagnetic fields on calcium dynamics in various cell types, including human leukocytes [34], growth hormone (GH)3 cells [35], and macrophages and lymphocytes [36]. The connection may be complex because of the differential effects of calcium ion influx in the vascular endothelium, which may trigger nitric oxide release and vasodilation, compared to the influx of calcium ions into vascular smooth muscle, which generally promotes vasoconstriction. Other aspects related to the role of calcium dynamics have been carefully researched and described [37,38]. However, it is unclear whether such cellular modulations of calcium dynamics might have been involved in the present findings since there have been limited studies on magnetic-related effects on calcium dynamics in either endothelial or vascular smooth muscle cells. However, a recent hypothesis suggests that static and time-varying magnetic field exposure may impact the frequency bands of calcium waves, thereby altering endothelial cell functions [39]. From a strictly hypothetical view and subject to further investigation, this concept might account for the previously observed actions of alternating magnetic fields on endothelial cell nitric oxide activity [40].

Finally, the observed 24.1% perfusion reduction and the corresponding reduction in PPG amplitude may, speculatively, have potential clinical implications. Because the present measurements were done on the hand, the most direct consideration would be for people with Raynaud’s condition, in whom a reduction of this amount might occur due to SR exposure, thereby triggering a flareup of the condition. A potentially more dangerous implication would be if such vasoconstrictive events were initiated in the coronary vasculature, especially in patients with compromised circulation. In such patients, such changes might be sufficient to trigger ischemic events. 

Study limitations

It is important to recognize that this was a pilot study to determine if there was any evidence of potential linkage between local hemodynamics and the SR frequency of 7.8 Hz. Some evidence consistent with this did emerge, despite using only six test subjects. Skin blood perfusion and PPG pulse amplitudes were statistically reduced after applying the alternating magnetic field. These findings are not conclusive but suggest that a more extensive probe into such interactions is warranted using more subjects. Furthermore, the present study applied the excitation with the magnet’s protective shell toward the dorsal aspect of the subject's palm, with the palm up. Accordingly, the excitation of the radial and ulnar arteries and the ulnar and median nerves depended upon the wrist girth, which was variable by subject. A recommendation for future studies would be to apply the field to the anterior surface. In the present study, the indication for a non-systemic effect was also judged based on the lack of change in the HRV. A recommendation for future studies would be to add localized simultaneous measurements to the non-treated limb. An additional limitation that should be considered is the absence of a sham control series.

## Conclusions

The preliminary results of this pilot study indicate that applying a local magnetic field at the SR frequency of 7.8 Hz decreases skin blood perfusion at the thenar eminence and reduces the finger PPG pulse amplitude on the same hand. There was no evidence of a systemic effect, as judged by an absence of changes in HRV. However, other possible systemic effects were not evaluated in this study and cannot be ruled out. These pilot findings lay the groundwork for a more extensive investigation of this process, which seems warranted based on the present findings. One area would be to extend the present work by including sham controls using a crossover design with a suitable number of subjects. Another area of interest would be to investigate, via retrospective literature or prospectively, if days or times of enhanced SR correlate with increased incidence of conditions exacerbated by increased vasoconstrictive processes. 
